# Potassium Iodide

**Published:** 1896-03

**Authors:** 


### Potassium Iodide.

Dr. Stengel administers potassium iodide as follows:

To two ounces of milk add two drachms essence of pepsin and one grain potassium iodide.

This forms a junket that effectually disguises the disagreeable taste of the iodide.—*Med. World.*

